# Cariprazine and clozapine: a systematic review of a promising antipsychotic combination for treatment-resistant schizophrenia

**DOI:** 10.1093/ijnp/pyaf053

**Published:** 2025-07-18

**Authors:** Sofia Pappa, Réka Csehi, Ellice Caldwell-Dunn, Zsófia Borbála Dombi, Stephan Hjorth

**Affiliations:** Department of Brain Sciences, Faculty of Medicine, Imperial College London, London, United Kingdom; West London NHS Trust, London, United Kingdom; Richter Gedeon Plc., Medical Division, Budapest, Hungary; West London NHS Trust, London, United Kingdom; Richter Gedeon Plc., Medical Division, Budapest, Hungary; Pharmacilitator AB, Vallda, Sweden

**Keywords:** partial agonist, psychopharmacology, combination treatment

## Abstract

Treatment-resistant schizophrenia affects over half of individuals with schizophrenia. Clozapine is the only approved treatment in the United States but often provides limited relief. Augmenting clozapine with cariprazine (CAR) (a D3-preferring dopamine D2-D3 partial agonist) may improve outcomes. This systematic review evaluated the efficacy and safety of this combination in patients with sub-optimal response. A search of PubMed, Embase, and Cochrane Library yielded 52 cases from 21 eligible studies to be included in the analysis. Patient and treatment characteristics and clinical outcomes were synthesized. Cariprazine replaced another antipsychotic or was added to clozapine monotherapy in 44.2% and 34.6% of cases, respectively. Before treatment, 90% of patients had positive and 81% had negative symptoms. Combination therapy improved these symptoms in 66% and 83% of cases, respectively. In 19 patients with Positive and Negative Symptom Scale scores available before and after treatment, total scores decreased by 43.4%, with positive and negative subscale reductions of 23.0% and 59.1%. The combination was generally well tolerated; some patients experienced weight loss and reduced clozapine-related side effects. New adverse events occurred in 19% (most commonly akathisia at 6%). Cariprazine was discontinued in 17% of cases due to side effects or lack of efficacy. Overall, the combination appears safe and promising, especially for persistent negative symptoms, likely due to complementary neuroreceptor effects. Larger controlled trials are needed to confirm these findings.

## INTRODUCTION

In an ideal pharmacotherapeutic scenario, schizophrenia would be managed with antipsychotic monotherapy using an agent that effectively addresses common symptom domains (eg, positive, negative, and cognitive symptoms) and demonstrates both safety and tolerability. Such an approach would ultimately improve the prognosis of the disorder while enhancing quality of life and patient adherence. Unfortunately, however, antipsychotic monotherapy rarely achieves these outcomes in real-world schizophrenia care.^1^

According to the National Institute for Health and Care Excellence (NICE) guidelines for the management of schizophrenia, at least two antipsychotics with different drug target profiles should be trialled first at optimized doses, for an adequate duration of time and with proper medication-adherence.^2^ If these antipsychotic trials fail, patients are categorized as having treatment-resistant schizophrenia (TRS),^3–6^ in which case clozapine monotherapy is recommended (at optimal dosage and adequate duration).^2^ In the event of poor response to clozapine monotherapy despite correct diagnosis, proper adherence, and monitored comorbid substance use, antipsychotic polytherapy could be considered.^2^

In fact, TRS is estimated to affect approximately 20% to 50% of patients with schizophrenia^3^^,^^6–8^ and presents a significant challenge in psychiatric care, including greater disease burden and poorer prognosis.^3^ Among pharmacological treatment options, clozapine stands out as still being the only medication recommended by the Food and Drug Administration^9^ for the pharmacological management of established cases of TRS. Despite its demonstrated superiority over other antipsychotics and real-world effectiveness in these patients,^3^^,^^10^ clozapine is also associated with serious adverse effects,^3^^,^^11^ thus it is not recommended as first-line treatment.^2^^,^^3^^,^^12^^,^^13^ Furthermore, studies indicate that 30% to 40% of patients do not achieve satisfactory symptom relief with clozapine alone,^14^^,^^15^ with some studies suggesting that this figure may even be as high as 60%.^16^ This high occurrence of clozapine-resistant schizophrenia (CRS) often leads clinicians to explore augmentation strategies, typically involving the addition of a second antipsychotic to enhance therapeutic outcomes.^17^

Evidence examining choice of augmentation agent to clozapine in CRS remains conflicting. A recent meta-analysis of randomized controlled trials examining clozapine augmentation strategies (including first- and second-generation antipsychotics risperidone, amisulpride, quetiapine, ziprasidone, sulpiride, sertindole, and pimozide), failed to demonstrate significant efficacy compared to placebo in positive and negative symptoms.^18^ Moreover, antipsychotic combinations comprising first- and second-generation antipsychotics may also raise a number of tolerability and safety issues.^19^ A retrospective chart review of lurasidone augmentation to clozapine suggests it may reduce illness severity and lead to overall improvement. However, observations of improvements in specific symptom domains were based on documentation in medical records rather than on scores from validated symptom scales and additional evidence from randomized controlled trials (RCTs) are currently not available.^20^ In this context, other meta-analyses suggest that dopamine receptor partial agonists (DRPAs) may offer particular benefit in mitigating the cardiometabolic risks of clozapine,^16^^,^^21–23^ in addition to reducing the risk of re-hospitalization in TRS patients.^24^ Overall, there is an urgent clinical need for novel treatment approaches, and support has been forthcoming for the usefulness of newer DRPAs in addressing limitations of current augmentation strategies in CRS.^25^

From a pharmacological perspective, combining agents with complementary pharmacodynamic and pharmacokinetic profiles is a key consideration in antipsychotic polypharmacy.^1^ For instance, some studies reported beneficial effects of aripiprazole, a dopamine receptor partial agonist, as add-on to clozapine.^1^^,^^26^^,^^27^ Given that cariprazine (CAR) also possesses strong partial agonist properties at D2 and, notably, an even higher affinity for D3 receptors^28^^,^^29^ and has shown efficacy against negative symptoms,^30–34^ it represents a pharmacologically rational augmentation strategy for clozapine, which has relatively weak interactions with these specific dopamine receptor subtypes.^1^ The complementarity in neuroreceptor target profiles achieved with clozapine and a dopamine receptor partial agonist, like CAR, could therefore offer a therapeutically advantageous approach to address the limitations of clozapine monotherapy in TRS, like for the alleviation of negative symptoms.

Cariprazine is a dopamine D2-D3 partial agonist antipsychotic with preferential binding to the D3 receptors.^28^^,^^29^ It has demonstrated efficacy in, and therefore received approval for the treatment of schizophrenia in the EU,^35^^,^^36^ and additional approval for the treatment of manic/mixed and depressive episodes associated with bipolar I disorder^36^ and major depressive disorder as adjunctive treatment to antidepressants by the Food and Drug Administration.^36^ Furthermore, the efficacy of CAR in various symptoms domains in schizophrenia, (eg, positive, negative, or cognitive symptoms,) has been shown in both clinical trials and real-world data.^30–34^^,^^37^^,^^38^ In fact, CAR is the only antipsychotic that has proven head-to-head efficacy over another antipsychotic, risperidone, in the treatment of predominant negative symptoms in schizophrenia patients.^31^ Moreover, evidence suggests that CAR is generally well tolerated, and may offer particular benefit for patients with cardiometabolic issues.^39^^,^^40^

To date, evidence regarding the safety and efficacy of CAR augmentation to clozapine derives from various case reports and case series, in addition to a pilot cohort study.^41^ The aim of this systematic review was to evaluate available evidence regarding the efficacy and safety of CAR–clozapine combination treatment, as well as to explore which specific patient populations might be likely to benefit from this combination and at what dosages. Finally, if found of promise, this systematic synthesis of the evidence could serve as a foundation for further research including in the form of a high-quality RCT.

## METHODS

### Search Strategy

This systematic review was conducted following the guidelines outlined in the Preferred Reporting Items for Systematic reviews and Meta-Analyses (PRISMA) statement.^42^ A comprehensive literature search was carried out across three databases (Embase, PubMed, and Cochrane Library), targeting peer-reviewed English-language articles published between January 2017 to October 2024. The search utilised the following terms: (cariprazin^*^) AND (clozapin^*^) AND (“case report^*^” OR “case report”/de OR “case stud^*^” OR “case study”/de OR “case seri^*^” OR “add-on” OR augmentation OR combin^*^). Additionally, hand searches were conducted and the reference lists of the included articles as well as earlier review articles were checked to uncover further relevant studies beyond those identified in the database search.

### Inclusion and Exclusion Criteria

Titles and abstracts of the articles from the database search were screened independently by two assessors (E.C.D. and Z.B.D.) to decide their eligibility for full-text revision. Full texts of the identified articles were subsequently reviewed independently according to inclusion and exclusion criteria. In case of disagreement, consensus was reached by further discussions involving the other authors.

The following inclusion criteria were applied during the screening process: (1) included studies must involve one or more adult human subjects, (2) the treatment must include CAR and clozapine combination treatment, and (3) the patient has a diagnosis of a schizophrenia spectrum disorder.

Articles were excluded if (1) CAR and clozapine were mentioned solely in the patient’s medical history, (2) there was insufficient information regarding the details of CAR-clozapine treatment, such as dosing strategy or timeline, and (3) the patient received CAR and clozapine only during the cross-titration period.

### Data Analysis

Data was collated on patient demographics (including sex, age, primary and secondary psychiatric diagnoses, medical comorbidities, and duration of illness), treatment characteristics (including previous and concomitant treatment, reason for augmentation, treatment length, and clozapine and CAR doses), symptom domains and side effects prior to CAR-clozapine treatment initiation.

Treatment efficacy was evaluated via objective clinical rating scales (Positive and Negative Symptom Scale, PANSS), where available, in addition to descriptive measures based on the clinical judgement of the treating clinician. Safety and tolerability were evaluated by impact on weight and BMI, in addition to the presence of adverse events during treatment.

## RESULTS

### Search Results

**Figure 1:**
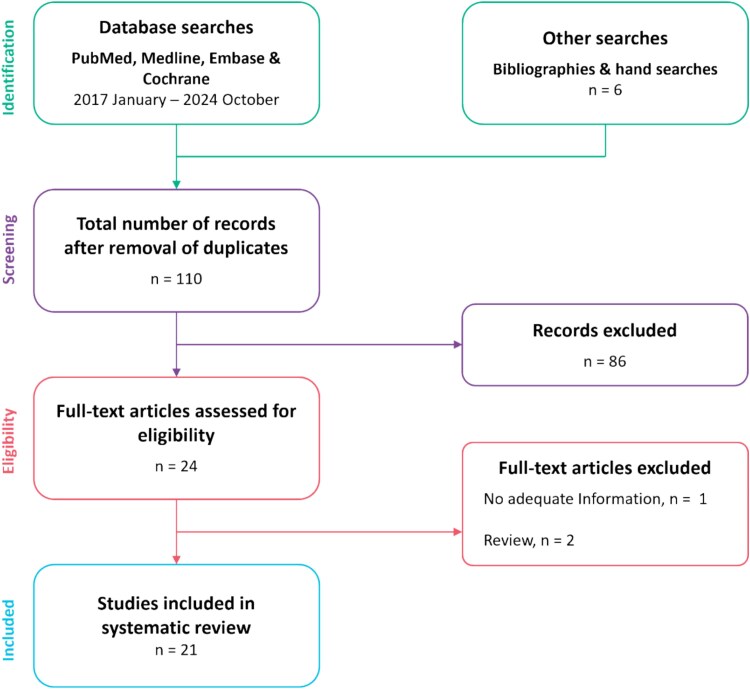
PRISMA flow chart.

Overall, 104 articles were retrieved via database search and 6 were found via hand search. No duplicates were detected. Based on titles and abstracts, 86 articles were excluded, leaving 24 articles for full-text assessment of eligibility. After reading the full text, a further 3 articles were excluded for the following reasons: inadequate trial information (*n* = 1) and review (*n* = 2). Overall, 21 articles were included in this systematic review, encompassing a total of 52 cases (Figure 1).

### Patient Characteristics

Total and individual patient characteristics are summarized in Table 1. Out of the 52 cases, 67% were male. The mean age was 37.7 years, and the average duration of illness was 13.8 years. The primary diagnosis was schizophrenia in 88%, and schizoaffective disorder in 12% of cases. Secondary diagnoses were reported in 21% of patients, including personality disorders (5.8%), polysubstance misuse (5.8%), cannabis use (3.8%), obsessive-compulsive disorder (3.8%), alcohol dependence (1.9%), social anxiety and phobias (1.9%) and depression (1.9%). Medical comorbidities were reported in 21% of patients, with obesity (9.6%), diabetes, and thyroid dysfunction (both 7.7%) being the most frequently reported physical conditions.

**Table 1:** Patient characteristics.

**Total**								
**Cases**	**Sex**		**Mean age (years)**	**Primary diagnosis**	**Secondary diagnosis** ^**a**^	**Medical comorbidities** ^**a**^	**Mean DOI**	**Reference**
52	67%	Male	37.7	88% Schizophrenia	5.8% Personality disorders	9.6% Obesity	13.8	N/A.
					5.8% Polysubstance misuse	7.7% Diabetes		
	33%	Female		12% Schizoaffective	5.8 % Substance use	7.7% Thyroid dysfunction		
**Individual**								
**Case**	**Sex**		**Age (years)**	**Primary diagnosis**	**Secondary diagnosis**	**Medical comorbidities**	**DOI (years)**	**Reference**
1	M		49	Schizophrenia	–	–	–	^43^
2	M		34	Schizophrenia	–	–	–	^44^
3	M		35	Schizophrenia	OCD	–	10	^45^
4	M		34	Schizoaffective disorder	–	–	1.5	^46^
5	F		Median age of patients 38 (27.5-45)	Schizophrenia	–	Obesity, hypertension, T1DM, hypothyroidism	15	^47^
6	M			Schizophrenia	–	–	14	
7	F			Schizophrenia	–	Obesity, diabetes	2	
8	M			Schizophrenia	–	Obesity, diabetes, dyslipidaemia, hyperuricaemia, SVAs	30	
9	M			Schizophrenia	–	–	20	
10	F			Schizophrenia	–	Obesity, diabetes, liver steatosis	22	
11	M			Schizophrenia	–	–	10	
12	M			Schizophrenia	–	–	5	
13	F			Schizophrenia	–	Osteoporosis, hyperparathyroidism	20	
14	M			Schizophrenia	–	Obesity	10	
15	F			Schizophrenia	–	–	18	
16	M			Schizophrenia	Polysubstance misuse	–	5	
17	M		30	Schizophrenia	–	–	11	^48^
18	F		41	Schizophrenia	–	–	13	^49^
19	M		45	Schizoaffective disorder	OCD, social anxiety, phobias	–	21	^50^
20	F		41	Schizophrenia	–	–	–	^51^
21	M		63	Schizophrenia	–	–	–	
22	F		34	Schizophrenia	Cannabis use	–	10	^52^
23	F		60	Schizophrenia	Depression, alcohol dependence	–	40	
24	M		23	Schizophrenia	Cannabis use	–	5	
25	M		51	Schizoaffective disorder	–	–	20	
26	M		28	Schizophrenia	Polysubstance misuse	–	10	
27	M		29	Schizophrenia	–	–	9	^53^
28	M		41	Schizophrenia	–	–	–	^42^
29	M		45	Schizophrenia	–	–	–	
30	M		47	Schizophrenia	–	–	–	
31	F		26	Schizoaffective disorder	–	–	–	
32	F		34	Schizoaffective disorder	–	–	–	
33	F		33	Schizophrenia	Personality disorder	–	–	
34	F		31	Schizophrenia	Personality disorder	–	–	
35	F		34	Schizoaffective disorder	Personality disorder	–	–	
36	M		37	Schizophrenia	–	–	–	
37	M		50	Schizophrenia	–	–	–	^54^
38	F		29	Schizophrenia		–	10	^55^
39	M		35	Schizophrenia	Polysubstance misuse	–	9	
40	M		31	Schizophrenia	–	–	9	^56^
41	M		48	Schizophrenia	–	–	–	^57^
42	M		25	Schizophrenia	–	–	8	^58^
43	F		20	Schizophrenia	–	–	3	^59^
44	M		23	Schizophrenia	–	–	7	^60^
45	M		27	Schizophrenia	–	–	12	
46	M		47	Schizophrenia	–	–	20	^61^
47	M		31	Schizophrenia	–	Past viral hepatitis	13	^62^
48	M		48	Schizophrenia	–	Hyperthyroidism	32	
49	M		36	Schizophrenia	–	–	23	
50	M		36	Schizophrenia	–	Diabetes mellitus	23	
51	M		34	Schizophrenia	–	Iron deficiency anemia, hiatus hernia, haemorrhoids, reactive airway disease	13	
52	F		25	Schizophrenia	–	Hypothyroidism, anemia	6	

Abbreviations: ASD, Autism spectrum disorder; DOI, Duration of illness; F, Female; M, Male; OCD, Obsessive compulsive disorder; SVA, T1DM, Type 1 Diabetes mellitus

a experienced by more than 5% of patients.

### Treatment Characteristics

Total and individual information regarding treatment characteristics is summarized in Table 2.

**Table 2:** Treatment characteristics.

**Total**								
		**Cariprazine – Clozapine treatment**				**Mean doses**		
**Cases**	**Psychiatric drug history** ^a^	Reason for augmentation	Treatment initiation	Concomitant medication(s)^b^	Mean treatment length (days)	Clozapine (mg)	Cariprazine (mg)	**REF**
52	38.5% amisulpride 38.5% olanzapine 34.6% risperidone 32.7% aripiprazole 23.1% haloperidol 11.5% quetiapine	**98.1%** NR **48.1%** SE	44.2% CAR replaced AP as adjunct to CLOZ 34.6% CAR added to CLO monotherapy 11.5% Unspecified 3.8% CLO replaced AP ad adjunct to CAR 3.8% CAR + CLOZ started concurrently 1.9% CAR added to CLOZ + adjunct AP	9.6% pregabalin 7.7% sertraline 7.7% lamotrigine 7.7% valproate 5.8% mirtazapine 5.8% biperiden 5.8% lorazepam 5.8% trazodone	157.8	369.6	**32.7%** 4.5 mg **26.9%** 3.0 mg **25%** 6.0 mg **13.5%** 1.5 mg **1.9%** Other	N/A


**Individual**								
		**Cariprazine – Clozapine treatment**				**Doses**		
**Case**	**Psychiatric drug history**	Reason for augmentation	CAR-CLOZ treatment initiation	Concomitant medication(s)	Treatment length (days)	Clozapine (mg)	Cariprazine (mg)	**REF**
1	amisulpride, aripiprazole	NR & SE	Clozapine + amisulpride switched to clozapine + cariprazine	sertraline, trazodone, lorazepam	25	200	1.5	^43^
2	–	NR	–	–	–	400	1.5 - 6.0	^44^
3	amisulpride, buspirone, clomipramine, haloperidol, olanzapine, paroxetine, risperidone, sertraline, valproate	NR & SE	Clozapine + amisulpride switched to clozapine + cariprazine	mirtazapine	61	100	6.0	^45^
4	bupropion	NR	–	aripiprazole	243	200	4.5	^46^
5	aripiprazole	NR & SE	Clozapine + aripiprazole switched to clozapine + cariprazine	sertraline	274	300	1.5 - 4.5	^47^
6	risperidone LAI	NR	Clozapine + risperidone LAI switched to clozapine + cariprazine	trazodone	304	200	3.0 - 4.5	
7	–	NR & SE	Cariprazine added to clozapine	pregabalin	243	300	3.0 - 4.5	
8	aripiprazole, haloperidol	NR & SE	Clozapine + aripiprazole + haloperidol switched to clozapine + cariprazine	biperiden, sertraline	122	500	3.0 - 4.5	
9	quetiapine	NR & SE	Quetiapine + cariprazine switched to clozapine + cariprazine	clonazepam, lamotrigine, pregabalin	122	375	1.5 - 4.5	
10	olanzapine	NR	Olanzapine + cariprazine switched to clozapine + cariprazine	lorazepam, trazodone	21	150	1. 5 - 6.0	
11	amisulpride, olanzapine	NR & SE	Clozapine + amisulpride + olanzapine switched to clozapine + cariprazine	alprazolam	61	400	1.5 - 6.0	
12	Amisulpride	NR & SE	Clozapine + amisulpride switched to clozapine + cariprazine	biperiden, lamotrigine, pregabalin	304	200	1.5 - 4.5	
13	Haloperidol	NR	Clozapine + haloperidol switched to clozapine + cariprazine	biperiden, lamotrigine, pregabalin	21	425	1.5 - 4.5	
14	Ziprasidone	NR & SE	Clozapine + ziprasidone switched to clozapine + cariprazine	estazolam	243	400	1.5 - 3.0	
15	Risperidone	NR	Clozapine + risperidone switched to clozapine + cariprazine	–	365	100	1.5 - 3.0	
16	Aripiprazole, lurasidone, risperidone	NR	Clozapine + aripiprazole, lurasidone, risperidone switched to clozapine + cariprazine	alprazolam, lamotrigine, pregabalin	456	200	1.5 - 4.5	
17	Flupentixol, olanzapine, paliperidone, quetiapine, risperidone	NR	Cariprazine added to clozapine + amisulpride	amisulpride	243	900	1.5 - 3.0	^48^
18	–	NR & SE	–	fluoxetine	–	175	4.5	^49^
19	Citalopram, lithium, nefazodone, perphenazine, reboxetine, risperidone, sertraline, valproate, venlafaxine, ziprasidone	NR & SE	Cariprazine added to clozapine	sertraline, valproate	335	450	4.5	^50^
20	amisulpride, aripiprazole, flupentixol, sertraline	NR & SE	Cariprazine added to clozapine	–	39	350	1.5 - 6.0	^51^
21	amisulpride, aripiprazole, haloperidol, sertraline	NR	Clozapine + amisulpride switched to clozapine + cariprazine	mirtazapine, pipamperone, pirenzepine, valproate	18	850	1.5 - 3.0	
22	amisulpride, aripiprazole, lurasidone, olanzapine, quetiapine	NR & SE	Cariprazine added to clozapine	pirenzepine	35	275	1.5 - 3.0	^52^
23	chlorpromazine, citalopram, fluphenazine, haloperidol, risperidone	NR	Cariprazine added to clozapine	valproate, venlafaxine	183	250	1.5	
24	aripiprazole, aripiprazole LAI, olanzapine, risperidone, paliperidone palmitate, haloperidol decanoate	NR & SE	Cariprazine added to clozapine	–	365	325	1.5	
25	amisulpride, olanzapine, quetiapine	NR	Cariprazine added to clozapine	mirtazapine	183	600	1.5	
26	amisulpride, aripiprazole, haloperidol, olanzapine, pipothiazine palmitate, quetiapine, risperidone, zuclopentixol decanoate, haloperidol decanoate	NR	Clozapine + amisulpride switched to clozapine + cariprazine	vortioxetine	183	700	1.5 reduced to every other day	
27	amisulpride, flurazepam, olanzapine, risperidone	NR	Cariprazine added to clozapine	–	183	800	1.5 - 3.0	^53^
28	–	NR & SE	Cariprazine added to clozapine	–	91	275	6.0	^42^
29	amisulpride	NR & SE	Clozapine + amisulpride switched to clozapine + cariprazine	–	91	300	4.5	
30	Lurasidone	NR	Clozapine + lurasidone switched to clozapine + cariprazine	–	91	650	4.5	
31	Aripiprazole	NR	Clozapine + aripiprazole switched to clozapine + cariprazine	–	91	200	3.0	
32	–	NR & SE	Cariprazine added to clozapine	–	91	200	1.5	
33	Amisulpride	NR	Clozapine + amisulpride switched to clozapine + cariprazine	–	91	200	6.0	
34	Aripiprazole	NR	Clozapine + aripiprazole switched to clozapine + cariprazine	–	91	200	6.0	
35	Amisulpride	NR	Clozapine + amisulpride switched to clozapine + cariprazine	–	91	500	1.5	
36	–	NR	Cariprazine added to clozapine	–	91	350	1.5	
37	Haloperidol, olanzapine	SE	Clozapine + cariprazine started concurrently	diazepam, trihexyphenidyl	183	37.5	3.0 - 6.0	^54^
38	Aripiprazole LAI, haloperidol, olanzapine, paliperidone, amisulpride	NR & SE	Clozapine + amisulpride switched to clozapine + cariprazine	–	243	400	1.5 - 3.0	^55^
39	Haloperidol, olanzapine, paliperidone LAI	NR & SE	Cariprazine added to clozapine	–	173	300	1.5 - 3.0	
40	Olanzapine	NR	Clozapine, olanzapine + amisulpride switched to clozapine, cariprazine + amisulpride	amisulpride	61	100	1.5 - 4.5	^56^
41	Amisulpride, aripiprazole, loxapine, olanzapine, risperidone	NR & SE	–	–	426	550	1.5 - 3.0	^57^
42	Aripiprazole, carbamazepine, chlorpromazine, haloperidol, lithium, olanzapine, risperidone	NR & SE	Cariprazine added to clozapine	lorazepam, promazine	183	600	1.5 - 6.0	^58^
43	Risperidone, quetiapine	NR & SE	Clozapine + cariprazine started concurrently	venlafaxine	30	100	3.0 - 6.0	^59^
44	–	NR	–	–	–	550	6.0	^60^
45	–	NR	-	–	–	350	6.0	
46	amisulpride, aripiprazole, olanzapine, paliperidone	NR	Cariprazine added to clozapine	–	–	400	4.5	^61^
47	olanzapine, risperidone, fluphenazine decanoate, amisulpride	NR	Cariprazine added to clozapine	–	365	400	3.0	^62^
48	olanzapine, risperidone, loxapine, amisulpride, aripiprazole	NR & SE	Cariprazine added to clozapine	–	30	550	3.0	
49	–	NR & SE	Cariprazine added to clozapine	sodium valproate	30	600	3.0	
50	Risperidone, amisulpride, fluphenazine decanoate, olanzapine	NR	Cariprazine added to clozapine	–	90	400	3.0	
51	Aripiprazole, olanzapine, risperidone	NR	Clozapine + olanzapine switched to clozapine + cariprazine	–	120	425	4.5	
52	Lurasidone, amisulpride, risperidone	NR	Clozapine, risperidone + amisulpride switched to clozapine + cariprazine	–	30	400	4.5	

Abbreviations: NR, non-response; SE, side-effect; REF, reference; LAI, long-acting injectable

a taken by more than 10% of patients

b taken by more than 5% of patients.

#### Psychiatric Drug History: Medications Used Prior to CAR–Clozapine Initiation

The majority of patients (82.7%) had been treated with at least one antipsychotic prior to commencing CAR–clozapine combination therapy (Table 2). More than half of the patients (53.8%) were reported as meeting the criteria for treatment resistance. Prior to starting CAR–clozapine combination treatment, the most commonly used previous antipsychotics were amisulpride, olanzapine (both 38.5%), risperidone (34.6%), aripiprazole (32.7%), haloperidol (23.1%), and quetiapine (11.5%).

#### Presenting Symptoms and Side-Effects Prior to Cariprazine–Clozapine Initiation

A combination treatment of CAR and clozapine was initiated in 98.1% of cases due to non or partial response to clozapine monotherapy or clozapine combination therapy with antipsychotics other than CAR (Table 2). Additionally, in half of the cases (48.1%), intolerable side-effects from previous treatment regimen further played a role in the initiation of CAR–clozapine combination treatment.

The most commonly reported symptoms prior to the initiation of CAR–clozapine combination treatment were positive symptoms (in 90.4% of patients) followed by negative (80.8%), affective (32.7%) and cognitive symptoms (19.2%), as well as impaired psychosocial/global functioning (23.1%) (Table 3). These percentages reported here are based primarily on descriptive clinical observations as standardized rating scales were often not used or not reported.

**Table 3:** Symptom characteristics before initiating cariprazine-clozapine combination and treatment outcomes.

**Case**	**Positive**		**Negative**		**Affective**		**Cognitive**		**Psychosocial / global functioning**		**Adverse events**	**Positive events**	**Discontinued**	**Reference**
	**Before**	**After**	**Before**	**After**	**Before**	**After**	**Before**	**After**	**Before**	**After**				
**1**	X	–	XX	–			X	–			Rhabdomyolysis		X SEs	^43^
**2**	X	↓	X	↓			X	↓						^44^
**3**	X	↓	X	↓								Weight loss Reduction in auto-aggressive compulsive behavior Reduction in motor disturbance Clozapine discontinued	X Monotherapy	^45^
**4**	X	n/a			X	n/a					Hyperactive delirium			^46^
**5**	X	↓	XX	↓	X	↓								^47^
**6**	X	↓	XX	↓	X	↓								
**7**			X	↓	X	↓								
**8**	X	–	XX	–	X	↓			X	↑				
**9**	X	↓	X	↓			X	–	X	↑				
**10**	X	–	X	–	X	–	X	–			Exacerbation of akathisia			
**11**	X	–	X	–	X	↑					Increased agitation			
**12**	X	↓	X	↓	X	↓	X	↓	X	↑				
**13**	X	↑	X	–	X	↑								
**14**	X	↓			X	–								
**15**	X	↓	X	↓								Reduction of dose of clozapine, reducing SEs		
**16**	X	↓	X	↓										
**17**	X	↓	X	↓			X	↓					X Non-response	^48^
**18**	X	–	X	↓	X	–			X	↑				^49^
**19**	X	↓	XX	↓	X	↓	X	↓	X	↑		Reduced obsessive compulsive thoughts Reduction of dose of clozapine, reducing SEs		^50^
**20**	X	↑			X	↑	X	↑			PISA syndrome		X SEs	^51^
**21**	X	–					X	↑			PISA syndrome Restlessness, pressured thinking		X SEs	
**22**	X	↓	X	↓					X	↑	Intrusive thoughts, resolved with dose reduction			^52^
**23**			X	↓								Weight loss		
**24**			X	↓										
**25**	X	–	X	↓	X	↓								
**26**	X	↑	XX	↓										
**27**	X	↓	X	↓	X	↓								^53^
**28**	X	↓												^42^
**29**	X	↓												
**30**	X	↓	XX	↓										
**31**	X	↓	X	↓							Dizziness at higher CAR doses			
**32**	X	↓	X	↓										
**33**	X	↓	X	↓										
**34**	X	↓	X	↓										
**35**	X	–	X	–									X Non-response	
**36**	X	–	X	–							Restlessness		X SEs	
**37**	X	↓	X	↓					X	↑		Weight loss Clozapine discontinued Reduction of akathisia	X Monotherapy	^54^
**38**	X	↓	X	↓					X	↑		Weight loss		^55^
**39**	X	↓	X	↓			X	↓				Weight loss		
**40**			X	↓	X	↓						Clozapine discontinued	X Monotherapy	^56^
**41**	X	↓	X	↓					X	↑		Reduction of dose of clozapine, reducing SEs		^57^
**42**	X	↓	X	↓					X	↑		Weight loss		^58^
**43**	X	↓			X	↓								^59^
**44**	X	↑	X	↓							Recurrent sexual fantasies, gambling, masturbation		X SEs	^60^
**45**			X	↓										
**46**	X	↓	X	↓					X	↑				^79^
**47**	X	↓	X	↓										^62^
**48**	X	↓	X	↓								Reduction of dose of clozapine, reducing SEs		
**49**	X	↓										Reduction of dose of clozapine, reducing SEs		
**50**	X	↓	X	↓					X	↑				
**51**	X	–											X Non-response	
**52**	X	–											X Non-response	
**Tot**	47 (90.4%)		42 (80.8%)		17 (32.7%)		10 (19.2%)		12 (23.1%)					
↓		31 (66%)		35 (83.3%)		10 (58.8%)		5 (50.0%)		12 (100.0%)				
↑		4 (8.5%)		0 (0.0%)		3 (17.6%)		2 (20.0%)		0 (0.0%)				
**-**		11 (23.4%)		7 (16.7%)		3 (17.6%)		3 (30.0%)		0 (0.0%)			12 (23.1%)	

Abbreviation: Tot: total

*Note.* X: presence of symptoms before cariprazine-clozapine combination treatment; ↑: increase in symptom (ie, worsening); ↓: decrease in symptom (i.e., improvement); -: no change in symptom.

In terms of tolerability issues prior to the augmentation of CAR–clozapine combination treatment (see Supplementary Material Table S1), the most commonly recorded side effects were weight gain (in 19.2% of patients), sedation (9.6%), extrapyramidal symptoms (EPS) (9.6%), hypersalivation (9.6%), tachycardia (7.7%), glucose dysregulation (7.7%), and constipation (5.8%). In 7.7% of the cases, side-effects were reported as a general statement, but remained unspecified.

#### Initiation of Cariprazine-Clozapine Combination Treatment

In most cases, CAR was either used to replace an antipsychotic previously given alongside clozapine (44.2%) or added to ongoing clozapine monotherapy (34.6%) (Table 2). In 11.5% of cases, the initiation method was not specified. Less commonly, clozapine replaced an *antipsychotic given in combination with CAR (3.8%), CAR and clozapine were started simultaneously (3.8%), or CAR was added to a regimen already including clozapine and another antipsychotic (1.9%).*

#### CAR and Clozapine Dosing and Combination Treatment Length

Dosing of clozapine and CAR, as well as combination treatment length are presented in Table 2. Most commonly, the maintenance dose of CAR was 4.5 mg/day (*n* = 17), followed by 3 mg/day (*n* = 14), 6 mg/day (*n* = 13), 1.5 mg/day (*n* = 7), and 1.5 mg/alternate days (*n* = 1). Clozapine maintenance dose ranged from 37.5 mg to 900 mg/day, with the most commonly prescribed maintenance doses of 200 mg/day (*n* = 9), 400 mg/day (*n* = 8), 300 mg/day, and 100 mg/day (both *n* = 4). The mean combination treatment length was 157.8 days.

#### Concomitant Medications during CAR–Clozapine Combination Treatment

During CAR-combination treatment, some patients received other concomitant medications (Table 2). The most common medications included including pregabalin in 9.6% of patients, sertraline, lamotrigine, and valproate in 7.7% of patients, as well as mirtazapine, biperiden, lorazepam, and trazodone in 5.8% of cases.

### Effectiveness of CAR–Clozapine Combination Treatment

#### Core Symptom Domains

Symptom improvements are summarized in Table 3.

Positive symptoms were the most commonly cited symptoms prior to initiation of clozapine–CAR treatment (*n* = 47/52, 90.4%). Of these 47 cases, CAR–clozapine treatment produced an overall satisfactory improvement in positive symptoms in 66% of cases, with 6 (12.8%) patients achieving complete remission of psychotic symptoms.^45^^,^^53^^,^^54^^,^^58^^,^^59^^,^^63^ In 23.4% of cases, positive symptoms remained unchanged, whereas in 8.5% of cases, positive symptoms were exacerbated during treatment.^45^^,^^57^^,^^59^^,^^62^

Negative symptoms were reported in 42/52 cases (80.8%). Of these, CAR–clozapine treatment yielded improvement in 83.3% of cases. In 16.7% of cases, negative symptoms were unchanged. There were no reports of worsening negative symptoms.

In addition to positive and negative symptoms, CAR–clozapine treatment produced positive effects on affective and cognitive symptoms. Where affective symptoms were reported (17 cases, 32.7%), treatment improved symptoms in 58.8% of cases. Affective symptoms worsened in 17.6% of cases in the form of heightened anxiety and dysphoria. Where cognitive symptoms were reported (10 cases, 19.2%), treatment improved symptoms in 50.0% of cases. In 3 cases, no change was reported, while in 2 cases, worsening of symptoms was recorded.

Where PANSS scores were available (*n* = 19) (Supplementary Material Table S2), CAR–clozapine treatment resulted in individual mean total % reduction in total PANSS scores by 43.41% [baseline mean (SD) 75.7 (32.3) vs post-treatment mean (SD): 44.3 (25.3)], positive PANSS scores by 23.0% [baseline mean (SD) 14.9 (8.9) vs post-treatment mean (SD) 9.7 (6.5), negative PANSS scores by 59.1% [baseline mean (SD) 23.9 (11.7) vs post-treatment mean (SD) 10.9 (7.8), and general psychopathology PANSS scores by 36.6% [baseline mean (SD): 37.6 (17.8) vs post-treatment mean (SD): 24.3 (13.8)].

In three cases, clozapine was gradually withdrawn and discontinued with continued use of CAR monotherapy with good symptomatic control.^48^^,^^53^^,^^63^

#### Global and Psychosocial Functioning

Where global functioning was reported (*n* = 12, 23.1%), CAR–clozapine treatment demonstrated favorable impact on functioning in all cases. Clinical Global Impression (CGI) scores were available for 15 cases. Within one case series (*n* = 12), post-treatment CGI-I scores were 2 (much improved) in 9 cases.^56^ A further three case reports indicated improvements in both CGI-S and CGI-I scores post-treatment.^47^^,^^53^^,^^56^^,^^58^ Furthermore, improvements in Global Assessment of Functioning (GAF) scores were noted in 3 cases.^50^^,^^54^^,^^63^ One case noted that improvements in GAF and Specific Level of Functioning scores were particularly evident at higher doses of 6 mg/day.^50^

Subjective improvements in global functioning included a wide range of activities, including social,^44^^,^^47^^,^^56^^,^^57^^,^^63^ occupational^44^^,^^52^^,^^58^^,^^63^ or recreational^44^^,^^52^^,^^54^^,^^55^^,^^63^ activities, as well as improved self-care.^47^^,^^57^

#### Other Symptoms

Three cases noted improvements in psychomotor drive.^44^^,^^45^^,^^48^ In a case of schizophrenia and OCD characterized by obsessive thoughts, auto-aggressive compulsive behavior and self-mutilation, CAR–clozapine treatment led to full control of auto-aggressive behavior, reduction in impulsivity, and an improvement in Yale-Brown Obsessive Compulsive Scale score from 22 to 3.^63^ In a further case of schizoaffective disorder with anankastia, CAR–clozapine treatment led to a reduction in obsessive thoughts from 2 hours to 30 minutes per day.^47^

#### Discontinuation of CAR

Overall, 9 out of 52 cases (17.3%) discontinued treatment with CAR. In four cases, combination treatment was discontinued due to poor treatment response^41^^,^^57^^,^^59^ and in 5 cases, due to tolerability issues, ie, rhabdomyolysis, Pisa syndrome, restlessness and gambling/recurrent sexual fantasies/masturbation.^41^^,^^43^^,^^49^^,^^60^ In three cases, clozapine was successfully withdrawn, and CAR was continued as monotherapy.^48^^,^^53^^,^^63^

Among the four cases that discontinued due to poor response, one involved a patient with schizoaffective disorder and comorbid personality disorder, where CAR treatment was stopped after 6 weeks.^41^ In two other patients, CAR augmentation did not yield sufficient symptom improvement after 3 months and 4 weeks, respectively, leading to discontinuation.^59^ Finally, in one case of schizophrenia, the patient—a CYP3A5^*^1/1^*^3 carrier, heavy smoker and regular coffee drinker—did not experience symptom relief until cimetidine was added to the regimen, which led to significant improvement in mental state and behavior.^57^

### Safety and Tolerability of CAR–Clozapine Combination Treatment

Overall, the addition of CAR was well tolerated and safe. In 5 cases, patients exhibited symptomatic improvements enabling the dose of clozapine to be reduced, thus improving side-effects, such as sedation, somnolence, constipation, hypersalivation, and nocturnal enuresis associated with the use of clozapine.^56^^,^^59^^,^^62^ Furthermore, weight loss was reported in 6 cases, 4 of them with recorded BMI reductions (30.5 to 26.3, 26.8 to 24.6, 28.4 to 25.6, and 29 to 27).^44^^,^^52–54^^,^^63^

Motor and neurological side effects developed in 5 cases: including exacerbation of akathisia,^56^ restlessness,^41^^,^^60^ agitation^56^ and the development of PISA syndrome.^60^ On the contrary, one case reported a reduction in EPS (acute dystonia, Parkinsonism, dysarthria, and akathisia) associated with previously used antipsychotics.^53^

A few patients experienced further adverse events. Dizziness emerged at higher CAR doses in one case,^41^ while in one case, self-reported intrusive thoughts emerged, however, it was managed by CAR dose reduction from 6 to 3 mg/day.^44^ One case developed disorganization and inappropriate behaviors on addition of CAR 6 mg/day, including recurrent sexual fantasies, increased masturbation and gambling.^43^ In one case receiving maintenance electroconvulsive therapy (ECT) in addition to CAR–clozapine, rhabdomyolysis developed during maintenance ECT sessions 25 days after CAR was started.^49^ In a case of schizoaffective disorder, hyperactive delirium developed after receiving one dose of zopiclone, when clozapine was prescribed in conjunction with CAR and aripiprazole.^51^

## DISCUSSION

To our knowledge, this is the first systematic review evaluating the effectiveness and tolerability of CAR–clozapine combination treatment. Overall, data from 52 patients with schizophrenia spectrum disorders were collected and analyzed, demonstrating favorable efficacy (particularly with regard to negative symptoms) and tolerability of CAR-clozapine combination treatment.

### Cariprazine-Clozapine Combination Treatment Efficacy

According to the findings of this systematic review, all patients had a diagnosis of a schizophrenia spectrum disorder (88% schizophrenia and 12% schizoaffective disorder). Previous medications included a variety of antipsychotics (eg, amisulpride, olanzapine, aripiprazole, and risperidone) and other psychotropic medication, including antidepressants and mood stabilizers. In the majority of cases, CAR replaced an antipsychotic previously used to augment clozapine or was added to augment clozapine monotherapy. Concomitant treatments included antidepressants, mood stabilizers, anxiolytics/sedatives or ECT. The maintenance doses ranged between 3 and 6 mg/day (with 4.5 mg/day being the most common) for CAR, and 100 to 400 mg/day (with 200 mg/day being the most common) for clozapine.

Within this systematic review, CAR–clozapine treatment proved particularly beneficial against negative symptoms, with 83.3% of those with negative symptoms exhibiting symptomatic improvement with combination treatment. The effect of combination treatment on positive symptoms was more variable; with 66% of cases with positive symptoms experiencing symptomatic improvement, while for a small proportion of patients (8.5%), positive symptoms worsened. Affective and cognitive symptoms were only reported in a smaller number of cases with mostly positive effects following the CAR–clozapine combination treatment.

**Figure 2:**
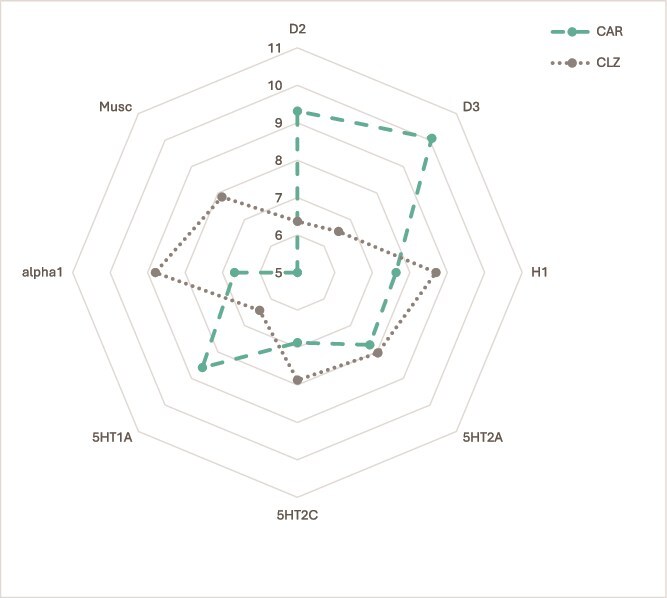
Neuroreceptor target profiles of cariprazine and clozapine. Dotted and dashed lines and dots represent drug profiles based on the 8 different targets depicted at the edges of the cobweb; affinity is highest at the edges, lowest at the center (0.1–10 000 nM log scale). The lines joining the dots represent the target profile of the agent with corresponding coding; the dotted line is clozapine (CLZ), and dashed line is cariprazine (CAR). Figure adapted from Hjorth, 2021.^1^

A recent article examining the target profile patterns of various antipsychotic drugs indicated that CAR and clozapine appear to have complementary neuroreceptor target profiles (Figure 2).^1^ It was highlighted that from a pharmacodynamics perspective, the strong partial agonist properties of CAR at the D2 and D3 receptors (and potentially at 5-HT1A) allow for a therapeutically beneficial combination by complementing clozapine’s relatively weak interactions with these targets.^1^ Notably, CAR can be an especially appealing option due to its high affinity for D3 receptors and its potential clinical efficacy in addressing primary negative,^31^^,^^46^ and cognitive^64^ symptoms, along with its extended pharmacokinetic half-life.^65^^,^^66^

In fact, effective treatment of negative symptoms is one of the most challenging aspects of schizophrenia management, with high disease burden. The aetiopathogenesis of negative symptoms includes evidence of reduced dopaminergic transmission within the prefrontal cortex and mesolimbic structures, accounting for diminished motivation and reward behaviors.^67^ Cariprazine exhibits a unique mode of action as the only antipsychotic that competes efficiently with dopamine at D3 receptors.^28^^,^^29^ One hypothesis for CAR’s pro-cognitive and anti-anhedonic effects is its partial agonist action at presynaptic D3 auto-receptors in the ventral tegmental area, disinhibiting dopamine release in the pre-frontal cortex,^68^ in addition to bringing accessory antidepressant properties via relatively high affinity and partial agonist action at serotonin 5HT1A receptors.^28^ In clinical studies, CAR is well-documented as being particularly effective against persistent negative symptoms, including within a systematic review of case studies (reducing negative symptoms in all cases),^32^ significantly reducing negative symptoms in comparison to placebo in pooled analyses from three clinical trials in acute exacerbations of schizophrenia^30^ and within observational studies.^33^ Moreover, CAR has been demonstrated to be the only antipsychotic significantly more effective than an active comparator, risperidone, in the treatment persistent negative symptoms.^31^

As a cautionary side-note worthwhile mentioning, some of the case reports listed cite concomitant treatment with amisulpride (eg, cases 17, 21, and 40).^48^^,^^57^^,^^60^ Combining amisulpride and CAR treatments is questionable from a pharmacodynamic point-of-view, as the former is a relatively high-affinity full antagonist of D2 and D3 receptors and thus may hamper the partial agonist action of CAR at these sites.

### Cariprazine-Clozapine Combination Tolerability

Cariprazine augmentation was overall safe and well tolerated with only 10 out of 52 cases (19.2%) reporting some type of adverse events within this systematic review. Of these, five were motor and neurological side effects, such as exacerbation of akathisia^56^ restlessness,^41^^,^^60^ agitation^56^ and the development of PISA syndrome.^60^ Indeed, DRPAs, including CAR, have been demonstrated to cause akathisia in both clinical trials and real-world settings.^32^^,^^39^ In CAR clinical trials, the median time to resolution of akathisia was 17 days when anti-akathisia medication was added, with 85% of events resolving, and 15 days in case of dose reduction of CAR with over 90% of events resolving as the emergence of akathisia is often dose dependent.^69^ To further minimize the risk of developing akathisia, it is also advised to consider adapting a slower up-titration strategy when introducing CAR.

Within one case, addition of CAR led to the development of recurrent sexual fantasies, masturbation and gambling. Comparable to DRPAs aripiprazole and brexpiprazole, CAR was hypothesised to be associated with (albeit rare) problems with impulse control.^70–72^ However, aripiprazole and brexpiprazole have received warnings from the FDA regarding the risk of impulse control disorders, while CAR has not.^71^ The emergence of this adverse event was suggested to be dose-dependent (as suggested in case of DRPA aripiprazole), although further evidence is needed to confirm this association.^60^ On the contrary, however, evidence suggests that CAR might improve auto-aggressive compulsive behaviors^63^ and obsessive-compulsive symptoms, as shown by case reports,^73^^,^^74^ a 12-week observational study^75^ and a pilot study of a 6-week double-blind, randomized, placebo-controlled study.^76^

Of other adverse effects noted within this systematic review, rhabdomyolysis has been previously reported with aripiprazole use within case reports, although the mechanism of action behind this is unknown.^77^^,^^78^

Despite these rare negative safety events, CAR augmentation was generally well-tolerated and led to discontinuation of CAR in only five patients (9.6%). Furthermore, the combination may offer particular benefit for those who experienced previous serious and/or distressing side effects of either clozapine or other augmenting agents. For example, the clozapine–CAR combination therapy was evidenced to cause a reduction in BMI in 6 cases,^44^^,^^52–54^^,^^63^ and improve symptoms to the extent that clozapine doses were able to be reduced in five cases, therefore reducing common side effects associated with clozapine.^56^^,^^59^^,^^62^ Notably, 3 patients were even able to discontinue clozapine and remain on CAR monotherapy, without symptomatic worsening.^48^^,^^53^^,^^63^

While clozapine and other antipsychotics are often associated with distressing side effects,^40^ including sedation, hypersalivation, and metabolic issues (significantly impacting patient adherence and quality of life), CAR’s low affinity for 5-HT2C, H1 and muscarinic receptors appears to reduce the risk of undesirable metabolic and cardiovascular side effects.^79^ This has been demonstrated by various randomised controlled clinical trials as well as by evidence from real-world settings.^32^^,^^39^ Therefore, by augmenting clozapine with CAR, clinicians can potentially mitigate these adverse effects while enhancing overall therapeutic outcomes.

### Clinical Implications

The findings of this review underline the need for clinicians to consider alternative treatment options for patients who do not achieve satisfactory outcomes with clozapine monotherapy or other augmentation strategies. Given that TRS and subsequently CRS affect a substantial proportion of individuals with schizophrenia, the introduction of effective augmentation strategies like CAR could significantly improve patient experience and care. Additionally, the complementary mechanisms of action between CAR and clozapine highlight the potential for personalised treatment plans tailored to individual patient profiles.

### Limitations and Future Research Directions

The limitations of this systematic review should be considered when interpreting its findings. Firstly, there is a potential for selection bias, as primarily positive reports are more likely to be written and published. Secondly, the variability in the included studies regarding patient characteristics, treatment protocols (including whether CAR and clozapine were started simultaneously or sequentially), and the timing of outcome assessments poses a limitation. Thirdly, most outcomes were narrative-based, often without specifying whether and which scales had been applied to assess symptom improvement. Using validated instruments—especially for negative symptoms—would strengthen future studies. Without this, positive results should be interpreted cautiously.

While preliminary evidence is promising, further research is necessary to validate these findings on a larger scale. Multi-center randomized controlled trials could provide more robust data regarding the efficacy and safety of CAR–clozapine combination treatment. Additionally, exploring optimal dosing strategies and identifying specific patient populations that may benefit most from this combination could enhance treatment precision.

## CONCLUSIONS

In conclusion, this systematic review suggests that CAR augmentation may offer a beneficial strategy for managing TRS spectrum disorders, with particular benefit in the case of persistent negative symptoms, or where previous augmenting agents or clozapine have caused serious side effects. By improving symptom control while maintaining a favorable safety profile, this combination therapy represents a hopeful advance in the pharmacological management of schizophrenia. Continued investigation will be crucial to fully understand its potential and refine treatment protocols for this challenging patient population.

## Supplementary Material

CAR-CLOZ_IJNP_Supplementary_material_REVISED_pyaf053

## Data Availability

The data supporting the findings of this study were obtained from previously published studies and publicly available sources. All relevant data have been included within the manuscript and supplementary materials. Additional details can be provided upon reasonable request.
