# Supplementary material for: Cariprazine and clozapine: a systematic review of a promising antipsychotic combination for treatment-resistant schizophrenia
Source: Int J Neuropsychopharmacol. 2025 Jul 18;28(8):pyaf053. doi: 10.1093/ijnp/pyaf053 (PMC12342903; doi:10.1093/ijnp/pyaf053)
Supplement: CAR-CLOZ_IJNP_Supplementary_material_REVISED_pyaf053 [file car-cloz_ijnp_supplementary_material_revised_pyaf053.docx]

Cariprazine & Clozapine:
A systematic review of a promising antipsychotic combination for treatment resistant schizophrenia

Sofia Pappa^1,2*^, Réka Csehi^3^, Ellice Caldwell-Dunn^2^, Zsófia Borbála Dombi^3^, Stephan Hjorth^4^

^1^Department of Brain Sciences, Faculty of Medicine, Imperial College London, London, UK

^2^West London NHS Trust, London, UK

^3^Richter Gedeon Plc., Medical Division, Budapest, Hungary

^4^Pharmacilitator AB, Vallda, Sweden

*Corresponding author:

Réka Csehi

Gyömrői street 19-21.

1103 Budapest

Hungary

csehir@gedeonrichter.com

+36 20 315 2072

**Supplementary Material – Table 1. Characteristics of side effects before initiating cariprazine-clozapine combination treatment**

| **SIDE EFFECTS** | | | | | | | | | | | | |
| --- | --- | --- | --- | --- | --- | --- | --- | --- | --- | --- | --- | --- |
| **Case** | **Unspecified** | **Weight gain** | **Sedation** | **Tachycardia** | **EPS** | **Hyper-prolactinaemia** | **Constipation** | **Nocturnal enuresis** | **Hyper-salivation** | **Sexual dysfunction** | **Glucose dysfunction** | **Reference** |
| **1** |  |  |  | X | X |  |  |  |  |  |  | ^58^ |
| **2** |  |  |  |  |  |  |  |  |  |  |  | ^54^ |
| **3** |  | X | X |  |  |  |  |  | X |  |  | ^43^ |
| **4** |  |  |  |  |  |  |  |  |  |  |  | ^61^ |
| **5** |  | X |  |  |  |  |  |  |  |  | X | ^52^ |
| **6** |  |  |  |  |  |  |  |  |  |  |  |  |
| **7** |  | X |  |  |  |  |  |  |  |  | X |  |
| **8** |  | X |  |  |  |  |  |  |  |  | X |  |
| **9** |  | X |  |  |  |  |  |  |  |  |  |  |
| **10** |  |  |  |  |  |  |  |  |  |  |  |  |
| **11** |  | X |  |  |  |  |  |  |  |  | X |  |
| **12** |  |  |  |  |  |  |  |  |  | X |  |  |
| **13** |  |  |  |  |  |  |  |  |  |  |  |  |
| **14** |  |  |  |  |  | X |  |  |  |  |  |  |
| **15** |  |  |  |  |  |  |  |  |  |  |  |  |
| **16** |  |  |  |  |  |  |  |  |  |  |  |  |
| **17** |  |  |  |  |  |  |  |  |  |  |  | ^50^ |
| **18** | X |  |  |  |  |  |  |  |  |  |  | ^57^ |
| **19** |  |  |  |  |  |  | X | X | X |  |  | ^53^ |
| **20** | X |  |  |  |  |  |  |  |  |  |  | ^60^ |
| **21** |  |  |  |  |  |  |  |  |  |  |  |  |
| **22** |  | X | X | X |  |  | X |  | X |  |  | ^55^ |
| **23** |  |  |  |  |  |  |  |  |  |  |  |  |
| **24** |  |  |  | X |  |  |  |  | X |  |  |  |
| **25** |  |  |  |  |  |  |  |  |  |  |  |  |
| **26** |  |  |  |  |  |  |  |  |  |  |  |  |
| **27** |  |  |  |  |  |  |  |  |  |  |  | ^44^ |
| **28** | X |  |  |  |  |  |  |  |  |  |  | ^41^ |
| **29** | X |  |  |  |  |  |  |  |  |  |  |  |
| **30** |  |  |  |  |  |  |  |  |  |  |  |  |
| **31** |  |  |  |  |  |  |  |  |  |  |  |  |
| **32** |  | X |  |  |  |  |  |  |  |  |  |  |
| **33** |  |  |  |  |  |  |  |  |  |  |  |  |
| **34** |  |  |  |  |  |  |  |  |  |  |  |  |
| **35** |  |  |  |  |  |  |  |  |  |  |  |  |
| **36** |  |  |  |  |  |  |  |  |  |  |  |  |
| **37** |  |  |  |  | X |  |  |  |  |  |  | ^45^ |
| **38** |  | X |  |  |  |  |  |  |  |  |  | ^56^ |
| **39** |  | X | X |  |  |  |  |  |  |  |  |  |
| **40** |  |  |  |  |  |  |  |  |  |  |  | ^51^ |
| **41** |  |  | X |  |  |  |  |  | X |  |  | ^49^ |
| **42** |  |  |  | X |  |  |  |  |  |  |  | ^46^ |
| **43** |  |  |  |  |  | X |  |  |  |  |  | ^47^ |
| **44** |  |  |  |  |  |  |  |  |  |  |  | ^59^ |
| **45** |  |  |  |  |  |  |  |  |  |  |  |  |
| **46** |  |  |  |  |  |  |  |  |  |  |  | ^81^ |
| **47** |  |  |  |  | X |  |  |  |  |  |  | ^48^ |
| **48** |  |  |  |  | X |  |  |  |  |  |  |  |
| **49** |  |  | X |  |  |  | X |  |  |  |  |  |
| **50** |  |  |  |  |  |  |  |  |  |  |  |  |
| **51** |  |  |  |  | X |  |  |  |  |  |  |  |
| **52** |  |  |  |  |  |  |  |  |  |  |  |  |
| **Total** | **4** | **10** | **5** | **4** | **5** | **2** | **3** | **1** | **5** | **1** | **4** |  |
| **%** | **7.7** | **19.2** | **9.6** | **7.7** | **9.6** | **3.8** | **5.8** | **1.9** | **9.6** | **1.9** | **7.7** |  |
| *X = marking the presence of the given symptom* | | | | | | | | | | | | |

**Supplementary Material – Table 2.** **Changes in** **PANSS scores throughout cariprazine-clozapine combination treatment**

|  | **PANSS Scores** | | | | | | | | | | | | | |
| --- | --- | --- | --- | --- | --- | --- | --- | --- | --- | --- | --- | --- | --- | --- |
| **Case** | **BASELINE**  **(PRIOR TO CARIPRAZINE-CLOZAPINE TREATMENT)** | | | | **POST-TREATMENT** | | | | | **CHANGES (%)** | | | | **Reference** |
|  | **Total** | **Positive** | **Negative** | **General** | **Total** | **Positive** | **Negative** | **General** | **Time of measurement (months)** | **Total** | **Positive** | **Negative** | **General** |  |
| **3** | 86 | 15 | 24 | 47 | 43 | 10 | 11 | 22 | **5** | -50.0 | -33.3 | -54.2 | -53.2 | ^43^ |
| **17** | 111 | 31 | 37 | 43 | 47 | 12 | 14 | 21 | **21** | -57.7 | -61.3 | -62.2 | -51.2 | ^50^ |
| **28** | 24 | 0 | 17 | 7 | 7  22  93  33  4  13  23 | 0 | 7 | 0 | **3** | -70.8 | 0 | -58.8 | -100.0 | ^41^ |
| **29** | 39 | 1 | 15 | 23 |  | 1 | 5 | 16 | **-** | -43.6 | 0 | -66.7 | -30.4 |  |
| **30** | 102 | 18 | 35 | 49 |  | 16 | 28 | 49 | **-** | -8.8 | -11.1 | -20.0 | 0 |  |
| **31** | 62 | 20 | 8 | 34 |  | 13 | 1 | 19 | **-** | -46.8 | -35.0 | -87.5 | -44.1 |  |
| **32** | 12 | 2 | 4 | 6 |  | 0 | 0 | 4 | **-** | -66.7 | -100.0 | -100.0 | -33.3 |  |
| **33** | 61 | 13 | 6 | 42 |  | 2 | 0 | 11 | **-** | -78.7 | -84.6 | -100.0 | -73.8 |  |
| **34** | 57 | 18 | 16 | 23 |  | 9 | 1 | 13 | **-** | -59.6 | -50.0 | -93.8 | -43.5 |  |
| **38** | 113 | 22 | 33 | 58 | 57 | 10 | 14 | 33 | **1** | -49.6 | -54.5 | -57.6 | -43.1 | ^56^ |
| **39** | 121 | 27 | 37 | 70 | 57 | 13 | 15 | 39 | **2** | -52.9 | -51.9 | -59.5 | -44.3 |  |
| **41** | 78 | 14 | 28 | 36 | 40 | 8 | 10 | 22 | **1** | -48.7 | -42.9 | -64.3 | -38.9 | ^49^ |
| **42** | 119 | 23 | 39 | 57 | 71 | 12 | 22 | 37 | **6** | -39.5 | -47.8 | -43.6 | -35.1 | ^46^ |
| **44** | 74 | 7 | 30 | 37 | 71 | 24 | 7 | 40 | **0.33** | -4.1 | 242.9 | -76.7 | 8.1 | ^59^ |
| **45** | 90 | 6 | 42 | 42 | 43 | 6 | 14 | 23 | **1** | -52.2 | 0 | -66.7 | -45.2 |  |
| **47** | 41 | 9 | 14 | 18 | 35 | 7 | 10 | 18 | **12** | -14.6 | -22.2 | -28.6 | 0 | ^48^ |
| **48** | 78 | 14 | 28 | 36 | 40 | 8 | 10 | 22 | **1** | -48.7 | -42.9 | -64.3 | -38.9 |  |
| **49** | 64 | 18 | 22 | 24 | 54 | 14 | 18 | 22 | **1** | -15.6 | -22.2 | -18.2 | -8.3 |  |
| **52** | 107 | 25 | 20 | 62 | 90 | 20 | 20 | 50 | **1** | -15.9 | -20.0 | 0 | -19.4 |  |
| **Mean**  **(SD)** | **75.7**  **(32.3)** | **14.9**  **(8.9)** | **23.9**  **(11.7)** | **37.6**  **(17.8)** | **44.4**  **(25.3)** | **9.7**  **(6.5)** | **10.9**  **(7.8)** | **24.3**  **(13.8)** |  | **-43.4**  **(21.7)** | **-23.0**  **(69.9)** | **-59.1**  **(27.6)** | **-36.6**  **(25.8)** |  |
| *PANSS, Positive and Negative Symptom Score* | | | | | | | | | | | | | | |
